# The Role of Adjuvant Radiation in the Management of Solitary Fibrous Tumors of the Central Nervous System: A National Cancer Database Analysis of 155 Patients

**DOI:** 10.7759/cureus.2656

**Published:** 2018-05-20

**Authors:** Nitesh Rana, Ellen Kim, Jerry Jaboin, Albert Attia

**Affiliations:** 1 Department of Radiation Oncology, Vanderbilt University Medical Center; 2 Radiation Medicine, Oregon Health & Science University

**Keywords:** solitary fibrous tumor, central nervous system, radiotherapy

## Abstract

Solitary fibrous tumors (SFT) are a rare neoplasm of mesenchymal origin. There is limited evidence on the epidemiology, treatment, and outcomes of SFT in the central nervous system (CNS). The National Cancer Database (NCDB) was queried for patients diagnosed with an SFT in the CNS as their only tumor diagnosis between 2003 and 2011. The final cohort included 155 patients who received surgery and had adequate information for analysis. Descriptive statistics, logistic regression, and Kaplan-Meier survival analyses were performed. Significance was calculated using a t-test, Fisher’s exact test, chi-square, log-rank test, or Cox model. Twenty-three patients (15%) underwent both surgery and adjuvant radiation while 132 (85%) underwent surgery alone. The treatment groups had comparable demographics and tumor size; median age 53 (range 25-80) and 11 females (48%) in the surgery and adjuvant radiation group, compared to 55 (20-89) and 71 (54%) in the surgery alone group, respectively. Radiotherapy methods included conventional and stereotactic dose and fractionations schemes. Information on margin status and re-resection rates was not available. No variables were significantly associated with receipt of adjuvant radiation. In single (p = 0.78) and multivariable (p = 0.86) survival analyses, the addition of adjuvant radiation did not significantly affect overall survival. Five-year overall survival was 88% with surgery alone versus 93% with adjuvant radiation. SFTs are rare neoplasms, especially in the CNS. Our study did not demonstrate an overall survival benefit for adjuvant radiation. The role of adjuvant radiation is still unclear and warrants further investigation.

## Introduction

Solitary fibrous tumors (SFTs) are a rare neoplasm of mesenchymal origin, which are molecularly characterized by inversion of chromosome 12q13, resulting in the unique fusion of the transcriptional repressor NAB2 gene with the transcriptional activator STAT6 gene [1-4]. SFTs were originally identified as arising from the pleura, but are now recognized to arise from extrapleural sites anywhere in the body. Hemangiopericytomas, previously thought to be a separate entity, were found to have identical histological findings as SFTs, as well as the NAB2-STAT6 gene, and are now considered as one entity within a spectrum of SFTs under the 2016 World Health Organization Classification of Tumors of the Central Nervous System [5]. SFTs occurring in the central nervous system (CNS) are an extremely rare occurrence that was first identified in 1996 [6]. Since then, there have been approximately 220 cases of CNS SFTs reported in the literature [7, 8].

The pre-operative diagnosis of CNS SFTs remains difficult as they appear similar to other intracranial soft tissue tumors radiographically. CNS SFTs clinically resemble meningiomas as they often originate from the dura, are usually well circumscribed, and often have a homogenous appearance [8, 9]. To obtain a definitive diagnosis, a surgical resection or biopsy is required. As such, the mainstay treatment for CNS SFTs is gross total resection, which is often the only treatment as the vast majority of SFTs are “benign” or indolent, with a low rate of local recurrence and almost no risk for extracranial metastases.

However, tumors that undergo a subtotal resection have a high risk of developing a local recurrence [8]. Additionally, SFTs that exist on the histologic spectrum of tumors previously classified as hemangiopericytomas are more aggressive with a high risk of developing local recurrence or extracranial metastasis [10-13]. Adjuvant radiation is often utilized to decrease the risk of recurrence. However, the role of adjuvant radiation therapy is unclear as there is limited evidence on the epidemiology, treatment, and outcomes of this disease. We have queried the National Cancer Database (NCDB) to evaluate the impact of radiotherapy in the treatment of the rare CNS SFTs.

## Materials and methods

The NCDB is a joint project of the Commission on Cancer of the American College of Surgeons and the American Cancer Society. The NCDB file was awarded to the authors for analytical purposes and reporting results to the NCDB are required, but the American College of Surgeons and the Commission on Cancer have not verified and are not responsible for the analytic or statistical methodology employed, or the conclusions drawn from these data by the investigator. The data used in the study are derived from a de-identified NCDB file that collects information on approximately 70% of all new invasive cancer diagnosis in the United States annually [14, 15]. This study used de-identified data and was exempt from institutional review board approval.

We extracted de-identified patient-level data for patients diagnosed with solitary fibrous tumor of the central nervous system (brain, cerebellum, cranial nerves, spinal cord, or meninges) between 2003 and 2011 from the NCDB. Of the 177 cases initially identified, <10 patients received no treatment or radiation alone, <10 patients had another tumor diagnosis, and <10 patients had inadequate treatment information for analysis. After applying the selection criteria, the final cohort consisted of 155 patients.

All available clinical information was extracted for each patient, including the following: demographics, Charlson-Deyo comorbidity index [16], SFT tumor parameters, treatment variables, and survival outcomes. Because of the relatively small cohort size, variables were grouped and frequently dichotomized to aid statistical analysis and to preserve patient confidentiality as required by the NCDB. Continuous variables were dichotomized after viewing distribution and to approximate 50% groups. Categorical variables were dichotomized logically also to keep groups with approximately even sizes. A category of <10 patients could theoretically be used to identify individual patients, and limiting report of small categories helps preserve patient confidentiality, as required in the NCDB data use agreement. The primary covariate of interest was treatment type. The primary outcome of interest was overall survival (OS), defined as the time from diagnosis to death or last contact (censored at that time). OS was estimated using the Kaplan-Meier method. Descriptive statistics, logistic regression, Kaplan-Meier survival analysis using the log-rank test for significance, and Cox proportional hazards multivariable analysis were performed using SAS v9.3 (SAS Institute, Cary, NC). Statistical significance was defined as a p-value of alpha <0.05 with a two-sided t-test, Fisher’s exact test, chi-square, or log-rank test.

## Results

**Figure 1:**
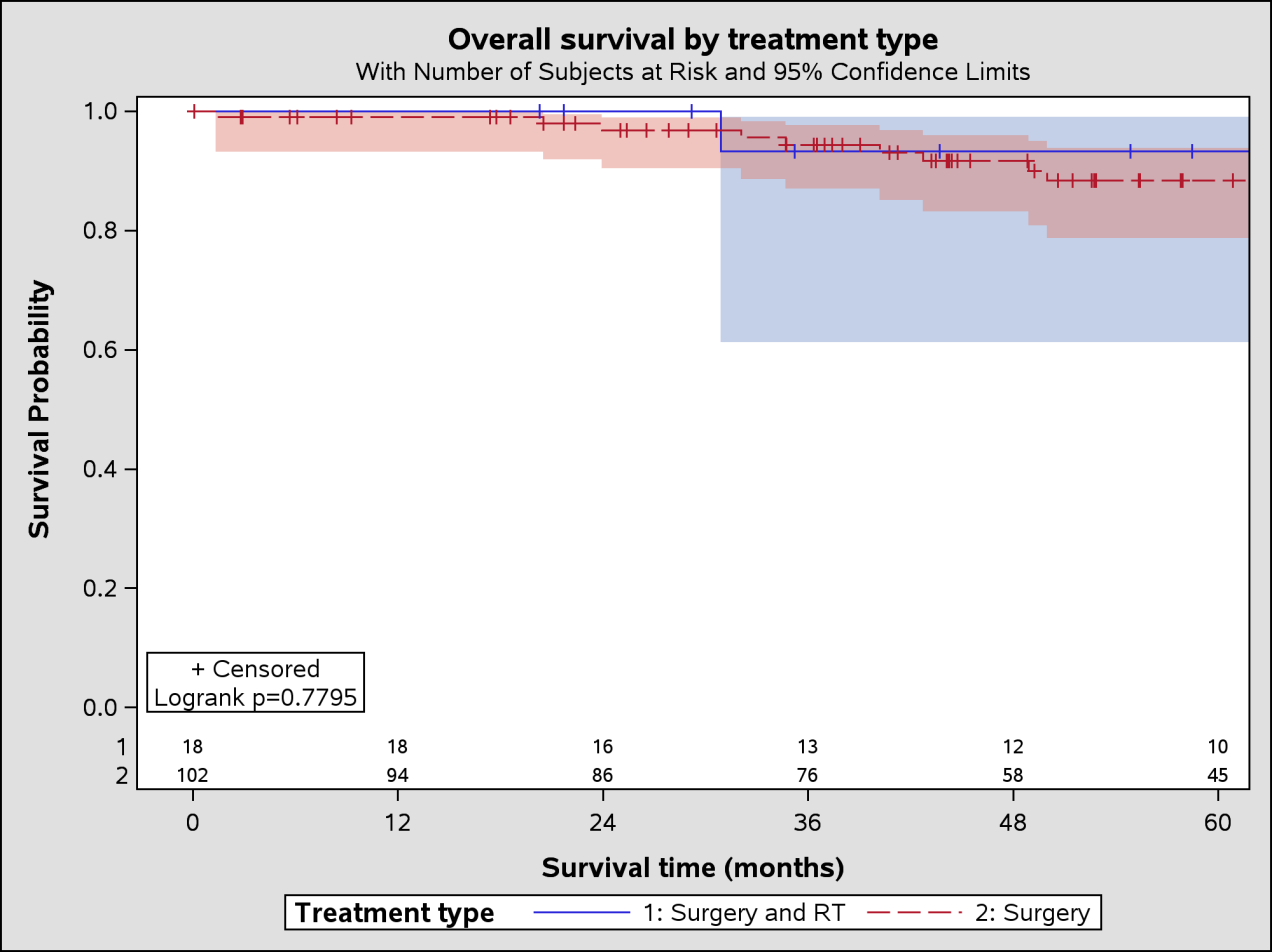
Kaplan-Meier overall survival estimates by treatment type.

Demographics, insurance, type of treatment facility, geographic region, regional income, education, and population were not significantly different between the treatment groups of surgery plus radiation compared to surgery alone (Table 1). Approximately half of the patients were men (p = 0.65) and approximately half were diagnosed at age <55 years (p = 0.82). Tumor size was comparable between treatment groups (p = 0.18); median for the surgery plus radiation and the surgery alone groups were 47 mm (range 33-71 mm) and 45 mm (range 6-150 mm), respectively. Kaplan-Meier OS estimates showed better survival with surgery and RT than with surgery alone (Table 2) but this was not statistically significant (p = 0.78, Figure 1). In multivariable analysis, there were no demographic or tumor characteristics that were significantly associated with receiving radiation therapy as well as surgery (Table 3). Most patients treated with radiation received intensity modulated radiotherapy (IMRT). Very few patients were treated with three-dimensional conformal radiotherapy (3D CRT). A majority of patients received 29-60 Gy (n = 20, 87%) and 27-34 fractions of radiation (n = 17, 74%). The vast majority of patients did not receive chemotherapy. Median follow-up was approximately 66 months for surgery plus radiation and approximately 53 months for surgery alone. Both treatment groups had a favorable OS. At five years, OS was 93% after receiving surgery plus radiation and 88% after surgery alone (p = 0.78).

**Table 1:** Baseline characteristics of patients by treatment. * Specific values not shown to protect patient privacy, as required by NCDB. NCDB: National Cancer Database

	Surgery and radiation N = 23	Surgery only N = 132	p-value
Gender: female	11 (48)	71 (54)	0.65
Gender: male	12 (52)	61 (46)	
Age: <55 yrs	12 (52)	65 (49)	0.82
Age: ≥55 yrs	11 (48)	67 (51)	
Insurance: other	<10 *	61 (46)	>0.05 *
Insurance: private	>13 *	71 (54)	
Regional income: lower	11 (48)	58 (44)	0.82
Regional income: higher	12 (52)	74 (56)	
Regional education: lower	11 (48)	57 (43)	0.82
Regional education: higher	12 (52)	75 (57)	
Population: lower	<10 *	57 (43)	>0.05 *
Population: higher	>13 *	75 (57)	
Facility: academic/research	11 (48)	58 (44)	0.82
Facility: other	12 (52)	74 (56)	
Region: central, other	11 (48)	67 (51)	0.82
Region: coast, atlantic	12 (52)	65 (49)	
Tumor size	Median 47, mean 48, range 33-71	Median 45, mean 44, range 6-150	0.18

**Table 2:** Kaplan-Meier overall survival (OS) estimate by treatment group.

	Surgery and radiation N = 23	Surgery only N = 132
1-year OS	100%	99%
2-year OS	100%	97%
3-year OS	93%	94%
4-year OS	93%	92%
5-year OS	93%	88%

**Table 3:** Multivariable analysis of baseline and tumor characteristics with receipt of radiation therapy.

Odds Ratio Estimates			
Factor	Point Estimate	95% Confidence Interval	
Male gender	1.227	0.482	3.125
Older age group	0.941	0.340	2.599
Non-private insurance	0.720	0.270	1.920
Higher regional income	0.821	0.286	2.357
Higher regional education	0.796	0.283	2.233
Higher population region	1.250	0.467	3.344
Year of diagnosis	0.977	0.828	1.152
Non-academic or research facility	0.880	0.329	2.351
Non-coastal region	0.862	0.328	2.268

## Discussion

Although extracranial SFTs are considered indolent tumors, CNS SFTs are known to behave more aggressively [17]. Additional factors that predict for worse outcomes include subtotal resection or positive surgical margins, high mitotic rate, tumor size, and tumor necrosis [18-20]. Unfortunately, we did not have information on all of these factors. Tumor size was not significantly associated with survival in our univariable analysis, though this may be in part due to low power.

There was inadequate information on the timing and sequence of radiation therapy and surgery. However since the diagnosis of SFT is extremely difficult without resection or biopsy, we assumed all patients received adjuvant radiation. Radiation treatments were also assumed to be delivered with a curative intent as number of fractions ranged from 27 to 34, which are typical for curative treatment. There may be a role for adjuvant radiation to improve outcomes for CNS SFTs. Radiation has been utilized for other benign and malignant CNS tumors, such as meningiomas, gliomas, and metastatic carcinomas, to improve local control after gross surgical resection. Moreover, with the advent of stereotactic radiosurgery and hypofractionated stereotactic radiotherapy, high doses of radiation can be delivered to the tumor bed while sparing radiation exposure to surrounding normal brain tissue, resulting in minimal treatment toxicities [21]. Unfortunately, the specific location of the tumor was not available. It is possible that the vicinity of the tumor to critical structures, such as the optic nerve or chiasm, brainstem, or eloquent regions, precluded the use of adjuvant radiotherapy. It is also possible that patients with a high grade and/or positive margin were more likely to have received adjuvant radiation than those with low grade, negative margin, or farther from critical structures. These factors could have reduced our ability to detect the effect of adjuvant radiation on survival. This is particularly important for small retrospective studies. The NCDB does not include detailed information about the radiation treatment plans such as dosimetry, which could have helped us try to control for this selection bias. Although margin status and grade are variables in the NCDB, the information on these variables was frequently missing in our cases. 

Other limitations of this study include small sample size, given the rarity of SFT, yet alone CNS SFTs. With such a rare histology, there is a concern for underdiagnosis or misdiagnosis which could impact the results of this paper. There may have been inaccurate reporting of tumor size and radiation doses to NCDB and very limited information in the database regarding the type of surgery performed, and gross total resection versus subtotal resection. Presumably, radiation therapy would be a greater benefit in tumors that underwent subtotal resection, recurrent disease, and had hemangiopericytoma variant or other aggressive histopathologic features. 

With a five-year overall survival of 88% with surgery alone, adjuvant radiation therapy may not be necessary in all CNS SFTs. Although radiation therapy was not found to impact survival in this study, we suspect that adjuvant radiation therapy plays an important role in improving local control as seen in the treatment of other malignant processes such as sarcoma and brain metastases. However, due to limited information from the NCDB on endpoints such as local failure, disease recurrence rates, side effects of treatments, and re-operation rates, the impact of radiation therapy on local control of SFT cannot be addressed.

The use of adjuvant radiation in CNS SFTs has been described in few case reports with excellent local control [22, 23]. In hemangiopericytomas, the biologically aggressive variant of SFTs, case series have reported improved local control, disease-free survival, and overall survival with adjuvant radiation [12, 24-27]. However, in this study, query of the NCDB failed to show a statistically significant improvement in overall survival with the addition of radiation therapy to surgery. The routine use of adjuvant radiation in CNS SFT should be determined in a multidisciplinary setting, on a case-by-case basis, where the extent of resection and grade as well as other important clinical factors would be taken into account.

## Conclusions

SFTs are rare neoplasms, especially in the CNS. This is the largest multi-institutional study of primary CNS SFTs to our knowledge. The primary treatment modality is surgery, with favorable five-year OS. Our study did not demonstrate a significant benefit in overall survival with adjuvant radiation. The role of adjuvant radiation is still unclear and warrants further investigation.
